# The microbiology and pathogenesis of nonfermenting Gram-negative infections

**DOI:** 10.1097/QCO.0000000000000969

**Published:** 2023-09-21

**Authors:** Vincenzo Di Pilato, Edward Willison, Anna Marchese

**Affiliations:** a Department of Surgical Sciences and Integrated Diagnostics (DISC), University of Genoa; b Microbiology Unit, IRCCS Ospedale Policlinico San Martino, Genoa, Italy

**Keywords:** nonfermenting Gram-negative bacteria, nosocomial infections, opportunistic pathogens, virulence factors

## Abstract

**Purpose of review:**

This review provides an overview of most recent evidence about pathogenesis traits and virulence factors contributing to successful colonization or infection by *P. aeruginosa*, *A. baumannii*, *S. maltophilia* and *B. cepacia* complex, among the most clinically relevant nonfermenting Gram-negative bacteria (NFGNB).

**Recent findings:**

The growing clinical importance of NFGNB as important opportunistic pathogens causing difficult-to-treat infections in a fragile patients’ population in stressed by numerous studies. Identification of novel virulence factors and deciphering of their mechanisms of action have greatly furthered our understanding of NFGNB pathogenesis, revealing that each pathogen-specific armamentarium of virulence factors (adhesins, motility, capsule, biofilm, lipopolysaccharide, exotoxins, exoenzymes, secretion systems, siderophores) can be likely responsible for the difference in the pathophysiology even in the context of a similar infection site. Emerging evidence of the immunomodulatory effect of some virulence factors is also acknowledged.

**Summary:**

NFGNB continue to be a serious global problem as cause of life-threatening opportunistic infections, owing to a highly heterogeneous content of virulence factors and their extensive number of intrinsic resistance mechanisms. Further efforts in development of novel effective antimicrobials and of alternative strategies targeting key virulence factors are warranted.

## INTRODUCTION

Nonfermenting Gram-negative bacilli (NFGNB) are a group of taxonomically heterogeneous aerobic, nonspore-forming bacilli that are unable to ferment carbohydrates and derive energy by using simple carbohydrates in an oxidative fashion [1]. These organisms are ubiquitously distributed in the environment as saprophytes, typically inhabiting moist ecosystems (e.g. water, soil, plants), and some are recognized as member of the healthy human gut microbiota [2–4].

Over past decades, however, NFGNB have emerged as important nosocomial pathogens, owing to their ability to persist in the hospital environment (e.g. sinks, respirators, nebulizers, dialysate, saline, catheters and other devices surfaces) and intrinsic antimicrobial resistance phenotypes (Table 1). As such, this group of microorganisms is increasingly recognized as cause of difficult-to-treat, life-threatening infections in fragile patients [e.g. neutropenic and intensive care unit patients, cystic fibrosis (CF)] [5], being a prevalent cause of nosocomial pneumonia and secondary bacteraemia (which can also develop from contaminated health-care equipment and surgical site infections) [6]. 

**Box 1:**
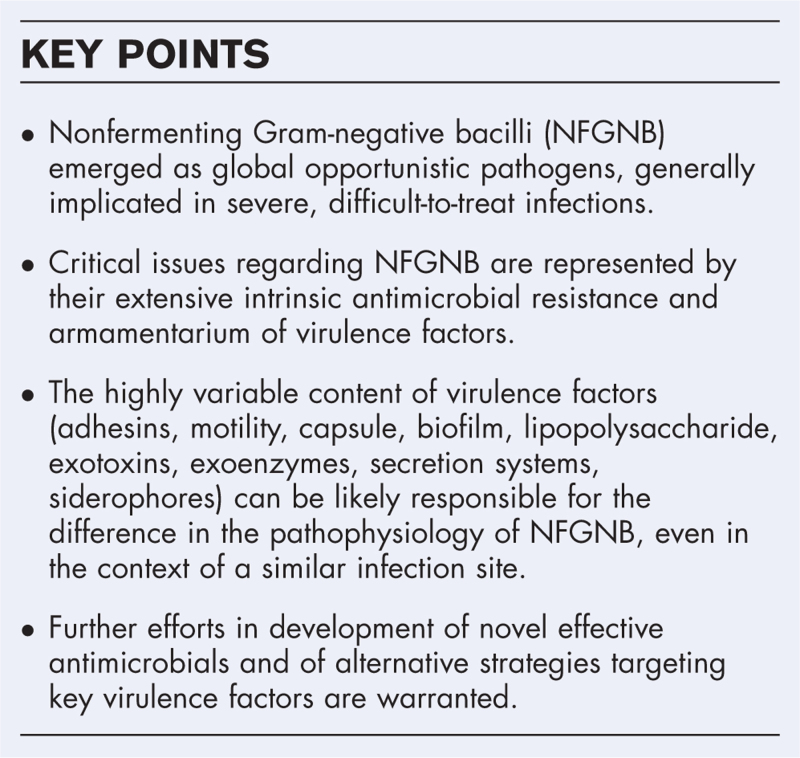
no caption available

**Table 1:** Overview of virulence factors and intrinsic antimicrobial resistance profiles of *P. aeruginosa*, *S. maltophilia*, *A. baumannii*, *B. cepacia complex*

	Adhesion	Motility	Toxicity	Invasion and survival	Secretion systems	Siderophores	Biofilm	Intrinsic reistance^a^
*P. aeruginosa*	- Type IV pili - Flagella	- Flagella	- ExoS - ExoT - ExoU - ExoY - exotoxin A - pore-forming toxon (EXIA) - pyocianin	- ExoY - PemA, PemB - haemolytic lipase - phospholipase C - leukocidin - lipoxygenase - alkaline protease - protease IV - elastases	- T2SS - T3SS	- pyocyanin - pyoverdine	- MucABCD - OMVs	- AMP, AMC, AMC, CTX, CRO, ETP, CM, TET, TIG, TMP, KAN
*S. maltophilia*	- SMF-1 fimbriae - Flagella	- Flagella	- LPS	- Flagella (evasion from host response) - protease - lipase - lecithinase - DNAs - Mucinase - Hyaluronidase			- SMF-1 fimbriae - Type IV pili - Diffusible Signal Factor (DSF)	- AMP, AMX, AMS, TIC, PIP, TZP, CRO, CTX, AZT, ETP, IMI, MEM, TMP, AGs, FOS, TET
*A. baumannii*	- Csu pili - O-polysaccharide - OmpA - Acinetobacter trimeric autotransporter (Ata) - biofilm-associated proteins (Bap*Ab*)	- Type IV pili - Surface-associated motility	- OmpA/CarO (Cell cytotoxicity) - OMVs (toxicity and inflammation)	- Capsule - OmpA, LPS (complement resistance) - Phospholipase C-E - Metalloendopeptidase	- T1SS - T2SS - T4SS - T5SS (Ata) - T6SS	- Acinetobactin, - Fimsbactin A–F - Baumannoferrin	- Csu pili - biofilm-associated proteins (Bap*Ab*)	- AMP, AMX, AMC, CTX, CRO, AZT, ETP, TMP, FOS, TET,
*B. cepacia complex*	- Cable pili - 22-kDa adhesin	- Flagella	- LPS (modified) - Hemolysin - Zinc metalloproteases	- Flagella - B. cepacia epidemic strain marker (BCESM	- T1SS - T2SS - T4SS - T6SS	- Salicyclic acid - Ornibactin - Pyochelin - Cepabactin		- AMP, AMC, AMS, TIC, TIM, PIP, TZP, CTX, CRO, AZT, ETP, CIP, CM, AGs, TMP, FOS, COL

AMP, ampicillin; AMX, amoxicillin; AMC, ampicillin/clavulanic acid; AMS, ampicillin/sulbactam; TIC, ticarcillin; TIM, ticarcillin/clavulanic acid; PIP, piperacillin; TZP, piperacillin/tazobactam; IMI, imipenem; MEM, meropenem; AZT, aztreonam; CTX, cefotaxime; CRO, ceftriaxone; ETP, ertapenem; CM, chloramphenicol; TET, tetracycline; TIG, tigecycline; TMP, trimethoprim; KAN, kanamycin; AGs: all aminoglycosides; FOS, Fosfomycin; CIP, ciprofloxacin; COL, colistin; OMVs: outer-membrane vescicles; LPS, lipopolysaccharide.

a *A. baumannnii*, *A. pittii*, *A. nosocomialis*.

Here, we provide an overview of the pathogenesis traits and virulence factors contributing to successful colonization or infection by *P. aeruginosa*, *A. baumannii*, *S. maltophilia* and *B. cepacia* complex, among the most clinically relevant NFGNB.

## TEXT OF REVIEW

### General concepts in infection pathogenesis and pathogenicity of nonfermenting Gram-negative bacteria

Following initial exposure, the earliest phase in the bacterial pathogenesis involves host cell- or tissue-specific interactions leading to colonization. A stabile adhesion to host cell receptors is fundamental to initiate that process, typically governed by production of fimbriae (or attachment pili) and of other surface adhesins (i.e. nonpilus, afimbrial adhesins) [7]. As an example, type IV pili (TFP) play a fundamental role as virulence factors in many species, being involved either in adherence or biofilm formation or motility [8,9,10]. The bacterial movement is generally mediated by flagella, specialized structures that aid in motility in viscous media or over surfaces (swarming), which can improve the infectious capabilities and the virulence potential of some species together with chemotaxis [11].

Following colonization, bacteria need nutrients (e.g. amino acids, iron) and appropriate environmental conditions (e.g. pH, oxygen) to grow, which means these factors can severely affect the establishment of an infection. Under permissive environmental conditions, secretion of toxic compounds (i.e. exotoxins) and/or extracellular enzymes damaging the mucosal integrity (e.g. hyaluronidases), or favouring evasion from the host's immune system [e.g. immunoglobulin A (IgA) proteases], can further contribute to progression of the infection and ultimately to a systemic dissemination [6].

Lipopolysaccharide (LPS) represents a major structural component of the outer membrane of Gram-negatives, playing a crucial role in the establishment of local and systemic inflammation processes. A large body of evidence showed that LPS can be involved in multiple steps of pathogenesis [12], since it is recognized by different host receptors [e.g. toll-like receptors (TLRs), patter recognition receptors (PRRs), and nucleotide-binding oligomerization domain-like (NOD-like) receptors], eliciting production of proinflammatory cytokines [13,14].

Some accessory structures can further participate in pathogenesis of NFGNB, like secretion systems, employed by most bacterial pathogens to mediate transport of various protein factors (e.g. adhesins, exotoxins, exoenzymes) onto the bacterial cell surface, into the surrounding environment, or directly into host cells, interfering with normal cellular functions and favouring the infectious process [15^▪▪^]. Most recently, it has been shown that Gram-negative bacteria can promote pathogenesis through secretion of outer-membrane vesicles (OMVs), spherical derivatives of the outer membrane filled with periplasmic content. OMVs represent a uniquely beneficial secretion option to deliver virulence factors, acquire nutrients, modulate the host immune system and produce biofilm [16,17].

Production of biofilm represents an additional strategy many bacteria can exploit to increase their pathogenicity [18,19^▪▪^]. During infections, indeed, biofilm can protect bacterial cells from phagocytosis, environmental stress, antibiotics, and dehydration [18]. Biofilm production strongly contributes to the survival in the hospital environment and on some medical devices, representing a major risk factor for hospital-acquired infections [20], and can concur to the exacerbation of chronic respiratory diseases [21,22]. Several reports indicate that proper biofilm development and maintenance is controlled via quorum sensing (QS), a specialized communication system relying on self-produced signalling molecules, known as autoinducers, acting as hormone-like compounds and controlling various metabolic processes [23,24^▪▪^].

#### Pseudomonas aeruginosa

The genus *Pseudomonas* includes hundreds of species characterized by a considerable phylogenetic variability, among which *P. aeruginosa* is of particular prominence as multidrug-resistant opportunistic human pathogen [25^▪▪^]; other species are rarely pathogenic in humans (e.g. *P. monteilii, P. mendocina, P. fulva, P. stutzeri*), but can represent either food/blood contaminants (e.g. *P. fluorescens* and *P. putida*) or important plant pathogens (e.g. *P. plecoglossicida*, *P. baetica*, *P. viridiflava*, *P. syringae*) [26].

*P. aeruginosa* can cause a wide range of acute and chronic opportunistic infections, particularly in patients with serious underlying diseases [e.g. immunodeficiency, CF, chronic obstructive pulmonary disease (COPD), ventilator-associated pneumonia, traumas] [27,28]. Attachment to the respiratory epithelium is mediated by flagellum, TFP (i.e. the most important adhesins of this pathogen) and outer membrane proteins [29,30]. Damages to host cells and tissues are exacerbated following delivery exotoxins via a type III secretion system (T3SS), a major virulence factor of *P. aeruginosa*[28,29,31], frequently associated with acute invasive infections and increased mortality (Table 1) [32,33,34].

At present, four exotoxins have been extensively characterized, including ExoS, ExoT, ExoU and ExoY [28,31,35]. While ExoT and ExoY have minor roles, ExoS and ExoU are considered the most clinically relevant toxins, although rarely expressed by the same isolate. Specifically, ExoS and ExoT can interfere with cell-to-cell adhesion, disrupting the actin cytoskeletal organization and inducing apoptosis [36,37]. ExoU is a potent phospholipase and is regarded as the most virulent T3SS effector, inducing rapid death of host cells owing to loss of plasma membrane integrity. ExoY acts as an immune response modulator, delaying activation of NF-κB and caspase-1, thus contributing to immune escape and survival within host infected tissues [38]. Recently, two novel effectors were described (i.e. PemA and PemB), thought to participate more in immune system evasion than in cytotoxic processes [39].

Several other exotoxins and exoenzymes have been recognized within the *P. aeruginosa* secretome, including: exotoxin A (ETA), inhibiting host protein synthesis; a pore-forming toxin (EXlA); an extracellular haemolytic lipase (LipA) degrading dipalmitoyl-phospatidilcholine (i.e. the major lung surfactant lipid); a phospholipase C (PLC) degrading surfactant and damaging cells; a leukocidin and a lipoxygenase (LoxA) interfering with the host lipid signalling resulting in host cell death; alkaline protease (AprA) damaging complement components and interferon (IFN)-α and IFN-γ; protease IV, degrading complement component as well as fibrinogen, lactoferrin, transferrin and elastin, an important component of respiratory tissue and vessel wall, additionally targeted by elasteses (LasA and LasB) (Table 1) [28,38,39].

Furthermore, the blue–green pigment pyocyanin, which gives *P. aeruginosa* colonies a typical colour, can mediate damages to host tissues via oxidative stress [40]. Interestingly, pyocyanin also serves as high-affinity iron chelating molecule, in association with pyoverdine (a second pigment), targeting transferrin and lactoferrin [41].

Concerning the host-microbe interaction, it was shown that LPS and flagellum can induce a TLR-4- and NF-κB-mediated inflammatory responses in the lung environment, respectively [28]. In CF patients, *P. aeruginosa* LPS can be recognized by the cystic fibrosis transmembrane conductance regulator (CFTR), mediating the activation of an inflammatory response via nuclear translocation of NF-κB [42], and can promote epithelial–mesenchymal transition in human bronchial epithelial cells together with the secreted protein PA 3611. Following this remodelling, pulmonary fibrosis is observed, representing a typical feature of chronic infections [43,44].

Production of biofilm is frequently observed owing to overproduction of exopolysaccharide alginate; this phenomenon also accounts for the conversion from the rough to the mucoid phenotype, posing the bases for the establishment of a chronic infection [43,45^▪^]. Such phenotypic switch aid *P. aeruginosa* in surviving in the lung microenvironment, decreasing bacterial clearance by the host immune system and effectiveness of antimicrobial agents, overall impairing the global host response to infection [46].

Conventionally, production of alginate is regulated by a complex regulatory network involving the *mucABCD* gene products, whose activity is regulated by AlgT (also known as AlgU or σ^22^ sigma factor); mutation affecting *mucA* have been shown to induce alginate synthesis and are frequently detected in mucoid isolates of *P. aeruginosa* from CF patients [46,47]. However, biofilm production also ranks among the processes finely controlled by QS (e.g. Ls, Rhl and Pqs) [48], together with production of pigments and secretion of OMVs (Table 1) [17,49].

Finally, another key feature strengthening the pathogenic potential of *P. aeruginosa* is represented by invasiveness [50]. In that regard, factors promoting dissemination from the lung into the bloodstream have been thoroughly investigated, and a model of disseminated infection involving the T3SS effector ExoS and T2SS LasB protease has been proposed. Once injected in neutrophils and in pneumocytes, ExoS can induce blockage of phagocytosis and compromission of the airway epithelial barrier, respectively. Differently, LasB can play a critical role in granting access to the bloodstream following cleavage VE-cadherins and disruption of the adherens junction of endothelial cells [50].

#### Stenotrophomonas maltophilia

The genus *Stenotrophomonas* contains more than 25 species (https://lpsn.dsmz.de/genus/stenotrophomonas), among which *S. maltophilia* emerged as crucial opportunistic pathogen responsible for fatal infections amongst highly debilitated patients, primarily involving the respiratory tract [51].

Although mechanisms underlying pathogenesis of *S. maltophilia* were not fully decoded, several studies shed light on certain peculiar features of this organism [52]. Adherence to human bronchial epithelial cells is primarily mediated by the characteristic SMF-1 fimbriae, which also participate in haemagglutination and biofilm formation (a critical factor for bacterial persistence on medical devices’ surfaces) [21]. Unlike environmental strains, SMF-1 fimbriae are regarded as core element of clinically derived strains of *S. maltophilia*, primarily form CF patients, strengthening the critical role these structures may have in stable colonization of the respiratory tract (Table 1) [53].

Adhesion processes were shown to be further supported by flagella, mediating interactions with host mucus membranes and synthetic surfaces in murine and *in vitro* models, respectively [54]. It should be also noted that motility functions mediated by flagella can aid in survival within host tissues, facilitating evasion from lysin, agglutinin, precipitin and other host humoral responses to infection [55], while their role appeared limited concerning biofilm production [56].

Rather, biofilm formation can be adjuvated by TFP, whose coding genes were identified by studies investigating the *S. maltophilia* genome diversity [55,56]. Production of biofilm represents a prominent feature of *S. maltophilia*[57,58^▪^], and several genes (e.g. *pgM, rmlA, rmlC, xanB, rpfF, bsmR, ax21*) and regulatory pathways (i.e. N-Acyl homoserine lactones [AHL], and the diffusible signal factor) were identified as critical factors for this process [59–62].

Other relevant virulence factors of *S. maltophilia* include LPS and production of exoenzymes. One study employing a rat lung model of infection demonstrated that mutant strains with limited LPS length were characterized by a significantly reduced virulence potential [63].

Production of multiple exoenzymes, like protease, lipase, and lecithinase, has been observed in clinical isolates from liver and trachea, but not in those recovered from blood (Table 1) [64]. Likewise, the StmPr1 protease encoding gene was unevenly detected among *S. maltophilia* isolates of clinical origin, being exclusively identified in isolates from persistent infections in CF patients, suggesting that some virulence traits may be a hallmark of strains causing chronic infections [53].

#### Acinetobacter baumannii

The genus *Acinetobacter* is highly diverse, containing hundreds of species (https://lpsn.dsmz.de/genus/acinetobacter), among which the majority are nonpathogenic environmental organisms. *A. baumanii* represents the most common species to cause infections followed by *A. calcoaceticus* and *A. lwoffii*, and possess more virulence potential than other *Acinetobacter* spp. [65].

Although *A. baumannii* has been long classified as nonmotile, due to lack of flagella, many strains are in fact motile, exploiting twitching motility or surface-associated motility [66,67]. While the former is driven by TFP [68], the latter is an appendage-independent form of movement, driven by the extrusion of extrapolymeric substances, and represents a key virulence factor of *A. baumannii* (Table 1) [69]. This type of motility can be regulated by QS and requires production of lipo-oligosaccahride, 1,3-diaminopropane, and of several proteins related to outer membrane, efflux pumps, and metabolism [66]. The QS system in *A. baumannii* has been shown to regulate a wide array of virulence mechanism, and basically relies on AbaI, a sensor protein that serves as autoinducer synthase to produce AHL, and on AbaR, its cognate receptor. As an example, following binding of AHL to AbaR, production of more AHL is triggered under a positive feedback loop, ultimately coordinating biofilm formation [70]. Establishment of a robust biofilm represents an advantageous survival mechanism of *A. baumannii*, explaining its propensity to cause infections associated with indwelling devices with high prevalence.

During early colonization, adherence functions appear to be driven by Csu pili, in association with production of exopolysaccharide; random mutagenesis experiments led to the identification of *csuE* as key gene in pilus and biofilm formation [71,72]. Other factors involved in adhesion include the antigenic O-polysaccharide (of LPS) and the outer membrane protein A (OmpA) [73]; the latter was additionally involved in biofilm production and complement resistance (Table 1) [74].

Using a murine pneumonia model, LPS was found to exert a major impact on the *A. baumannii* virulence, conferring resistance to normal human serum and triggering production of proinflammatory cytokines [e.g. interleukin (IL)-8, TNF] via stimulation of TLR4 signalling [75]. As a consequence of a TLR4-mediated cytokine storm, a sepsis syndrome may follow [76].

Of note, cytokine production was found to be stimulated also via TLR2, although molecular mechanisms were not elucidated yet [76]. Beyond the immunostimulatory effect of LPS, capsule has been also regarded among prime virulence factors, mediating evasion from host innate immune response (e.g. complement, phagocytosis).

Several other virulence factors can overall contribute do *A. baumanni* pathogenicity (Table 1), including: i) production of OMVs; ii) exoenzymes (e.g. outer membrane protein phospholipases C and E, metallo-endopeptidase); iii) siderophores (e.g. acinetobactin, fimsbactin A–F, baumannoferrin); iv) a high-affinity zinc acquisition system (i.e. ZnuABC) [77,78].

#### *Burkholderia cepacia* complex

The *Burkholderia cepacia* complex (Bcc) consists of several species of closely related and extremely versatile organisms (i.e. *B. cepacia, B cenocepacia, B. multivorans, B. stabilis, B. vietnamiensis, B. solosa, B. Ambifaria, B. anthina, B. pyrrocinia, B. ubonensis*) that occur in several natural environments [79]. However, individual members of this complex can cause opportunistic nosocomial infections, especially in CF patients (as causative agent of the cepacian syndrome [80^▪^]) and immunocompromised individuals.

Due to a defective mucus clearance, indeed, the CF lung represents an extremely favourable environment for colonization. Furthermore, CF airways epithelial cells showed an increased expression of the cytokeratin 13 intermediate protein, a substrate that Bcc cable pili (which also aid in cell aggregation) together with and 22-kDa associated adhesin can recognize as host receptor, thus granting optimal binding properties (Table 1) [3].

The Bcc LPS structure represents another prominent virulence factor, since it markedly differs from that of other Gram-negatives, in that it contains less phosphate and 3-deoxy-d-manno-oct-2-ulosonic acid (KDO), together with a modified lipid A backbone. Such a molecular configuration was found to overall lower the anionic charge of the cell surface and alter the effectiveness of cationic antimicrobial peptides and polymyxin [81]. Noteworthy, the endotoxic potential of Bcc LPS was markedly higher than that of other CF pathogens (e.g*. P. aeruginosa*), triggering an increased neutrophils activity and IL-8 production from epithelial cell [82,83].

Other notable virulence factors include: flagella, that can contribute to invasion of lung epithelial cells, but not to adherence [84]; biofilm production, mostly from *B. cenocepacia* and *B. multivorans*; production of siderophores, including salicyclic acid, ornibactin, pyochelin, cepabactin; exoenzymes (zin metalloproteases) and toxins (haemolysin) improving invasive properties; and the *B. cepacia* epidemic strain marker (BCESM), a pathogenicity island encoding a second QS system and adjunctive metabolic functions in epidemic strains [85]. Interestingly, the co-occurrenceof cable pili, the 22-kDa adhesin and of BCESM, has been regarded as a key feature of hypertransmissible Bcc (epidemic) strains [3].

## CONCLUSION

Globally, *P. aeruginosa, A. baumannii*, *S. maltophilia* and the *B. cepacia* complex rank amongst the most clinically relevant NFGNB, which have all evolved a highly heterogeneous content of virulence factors and accumulated an extensive number of intrinsic antimicrobial resistance mechanisms (Table 1), making evident the success of these opportunistic pathogens. As future lookout, the development of novel effective antimicrobials is urgently needed to counteract the emergence of difficult-to-treat NFGNB. In parallel, a genome-based understanding of molecular mechanisms underlying the pathogenicity and virulence traits might foster the rational design of alternative strategies to combat infections caused by NFGNB.

## Acknowledgements

*None.*

### Financial support and sponsorship

*This work was not supported by any funding.*

### Conflicts of interest

*There are no conflicts of interest.*

## REFERENCES AND RECOMMENDED READING

*Papers of particular interest, published within the annual period of review, have been highlighted as:*

▪ of special interest
▪▪ of outstanding interest
